# A hybrid cloud load balancing and host utilization prediction method using deep learning and optimization techniques

**DOI:** 10.1038/s41598-024-51466-0

**Published:** 2024-01-16

**Authors:** Sarita Simaiya, Umesh Kumar Lilhore, Yogesh Kumar Sharma, K. B. V. Brahma Rao, V. V. R. Maheswara Rao, Anupam Baliyan, Anchit Bijalwan, Roobaea Alroobaea

**Affiliations:** 1 Department of Computer Science and Engineering, Chandigarh University, Gharuan, Mohali, Punjab, 140413 India; 2 Department of Computer Science and Engineering, Koneru Lakshmaiah Education Foundation, Greenfield, Vaddeswaram, Guntur, AP India; 3 Department: Computer Science and Engineering, Koneru Lakshmaiah Education Foundation, Vaddeswaram, Andhra Pradesh India; 4 Department of Computer Science and Engineering, Shri Vishnu Engineering College for Women(A), Bhimavaram, India; 5 Arba Minch University, Arba Minch, Ethiopia; 6 Department of Computer Science, College of Computers and Information Technology, Taif University, P. O. Box 11099, 21944 Taif, Saudi Arabia

**Keywords:** Engineering, Mathematics and computing

## Abstract

Virtual machine (VM) integration methods have effectively proven an optimized load balancing in cloud data centers. The main challenge with VM integration methods is the trade-off among cost effectiveness, quality of service, performance, optimal resource utilization and compliance with service level agreement violations. Deep Learning methods are widely used in existing research on cloud load balancing. However, there is still a problem with acquiring noisy multilayered fluctuations in workload due to the limited resource-level provisioning. The long short-term memory (LSTM) model plays a vital role in the prediction of server load and workload provisioning. This research presents a hybrid model using deep learning with Particle Swarm Intelligence and Genetic Algorithm (“DPSO-GA”) for dynamic workload provisioning in cloud computing. The proposed model works in two phases. The first phase utilizes a hybrid PSO-GA approach to address the prediction challenge by combining the benefits of these two methods in fine-tuning the Hyperparameters. In the second phase, CNN-LSTM is utilized. Before using the CNN-LSTM approach to forecast the consumption of resources, a hybrid approach, PSO-GA, is used for training it. In the proposed framework, a one-dimensional CNN and LSTM are used to forecast the cloud resource utilization at various subsequent time steps. The LSTM module simulates temporal information that predicts the upcoming VM workload, while a CNN module extracts complicated distinguishing features gathered from VM workload statistics. The proposed model simultaneously integrates the resource utilization in a multi-resource utilization, which helps overcome the load balancing and over-provisioning issues. Comprehensive simulations are carried out utilizing the Google cluster traces benchmarks dataset to verify the efficiency of the proposed DPSO-GA technique in enhancing the distribution of resources and load balancing for the cloud. The proposed model achieves outstanding results in terms of better precision, accuracy and load allocation.

## Introduction

Cloud computing enables the optimum utilization of computing resources using its dynamic service model. Cloud services require adaptive distribution of computing resources with dynamic resource scalability, which helps in delivering a Quality-of-Service (QoS) with the most minor resource expenses. However, in complicated cloud settings with changing workloads, it might be challenging to implement dynamic resource distribution for heterogeneous applications^1^.

Cloud storage solutions have seen a considerable increase in demand since the emergence of the expanding IoT with Industry 4.0 technology regulations for various data-processing activities such as storage spaces, searching for resources, and mapping. The cloud is linked with IoT-enabled applications across multiple industry verticals, which helps them to utilize computing resources from remote locations. Cloud computing represents a pay-per-use mode of offering computing resources accessible on-demand from hosting companies^2^.

Cloud computing is becoming incredibly important in the educational and IT sectors and everyday life because of many characteristics, including no initial cost, immediate customer service, reliability, adaptability, and simple accessibility. The phrases Platform as a service (PaaS), Software as a service (SaaS), Data as a Service (DaaS) and Infrastructure as a service (IaaS) are utilized in the solutions offered by organizations such as Google, Microsoft, Amazon, and many other individuals, for devices, infrastructure, software, applications, and any technology. Clients of cloud-based services can utilize them from anywhere, on any device, and at any time. The cloud-computing allows the client to access its resources or programs according to their specific requirements^3^.

Many data centers have been established worldwide due to the growing acceptance of cloud technology. These data centers can offer various computing services, such as storage spaces, Networks, servers, and software for Industry, e-commerce, and other online uses. A novel cloud computing architecture mainly includes significant computing resources^4^. Cloud computing services primarily rely significantly on its data center. Because these data centers consist of different kinds of technology, including servers and storage spaces, they represent a significant cause of global power consumption. In addition, it is anticipated that the power consumed by data centers worldwide rise by 70% because of the ongoing proliferation of cloud services. To accomplish long-term growth, it has become necessary to utilize environmentally friendly computing strategies and reduce data center's energy utilization due to their substantial power use, rapid rate of expansion, and cumulative environmental footprint^5^.

This issue can be reduced with precise forecasting of the potential workload behaviour for resources via accurate observation. Effective monitoring and keeping a record of how much time and effort are expended on various resources, such as memory, central processing unit, space for storage, and the bandwidth of the network, helps solve this issue. These hints of previous use can then be examined and used in predictive modelling. This sense of anticipation is essential to provision resources in the cloud^6^ properly. The primary strategies for increasing resource utilization and reducing excessive provision issues include virtualization, Virtual machines and dynamic workload. Virtualization enables individual physical devices (PMs) to be split into multiple identical VMs, which allow the exploitation of different physical capabilities^7^.

The remaining benefits of virtualization include better server management, improved resource utilization, and cost-effective data center architecture. But when the server systems aren’t in execution, their resources, such as power and energy, are squandered, and consequently, data center incompetence occurs through idle power consumption. An excellent way to cut down on the electricity and energy used by data centers is to consolidate machines and virtual servers. The VMs are combined over PMs, so no additional PMs need to be installed^8^. In massive operations, integration of VMs and computing resource allocation is essential as groups of machines address complex optimization issues. A cloud computing system must utilize all of its capacities to the most significant potential to satisfy the rising demand for its products and services.

A further considerable symmetry concern must be addressed: higher and lower oscillations within cloud workloads. Any excessive allocations of computing resources might increase energy consumption and raise expenses. In existing research, cloud computing has widely utilized deep learning and machine learning methods for load balancing. Some research optimization-based techniques are also used in VM machine and resource mapping^9^. The critical contribution of the study is as follows:

- This research presents Deep learning with Particle Swarm Intelligence and Genetic Algorithm based “DPSO-GA”, a Hybrid model for dynamic workload balancing in cloud computing.
- A PSO method also helps to fine-tune the Hyperparameters. The proposed model integrates the resource utilization in a multi-resource utilization array, which helps to overcome the load balancing and over-provisioning issues.
- The proposed hybrid model is divided into two modules. The first phase utilizes a hybrid PSO-GA approach to address the prediction challenge by combining the benefits of the two methods.
- In the second phase, CNN-LSTM is utilized. Before using the CNN-LSTM Approach to forecast the consumption of resources, a hybrid approach, PSO-GA, is used for training it. In the proposed framework, a one-dimensional CNN and LSTM are used to forecast cloud resource utilization at various subsequent time steps.
- Comprehensive simulations are carried out utilizing the RUBiS and Google cluster datasets to verify the efficiency of the proposed DPSO-GA technique in enhancing the distribution of resources and load balancing for the cloud.
- To the best of our knowledge, this is the first attempt to utilize the fusion of deep learning (CNN-LSTM) and optimization techniques (PSO-GA) for workload prediction and load balancing in the cloud.

The complete article is organized as follows: Section two covers a literature review on cloud computing, load balancing, deep learning and optimization methods in load balancing and their challenges. Section three presents materials and techniques which offer the proposed model's working, design, procedures, and parameters. Section four covers the experimental results and comparison of the proposed model and existing solutions; this section also covers a discussion subsection. Section five covers the conclusion and future direction of the research, limitations and critical aspects.

## Literature review

The difficulties of consolidating cloud data centers need to be studied. Combining VMs is possible through virtualization, which improves resource utilization and reduces energy use. Cloud service providers build many virtual machines on one physical Host. The newest energy-efficient virtual machine integration techniques for data storage processors, including data centers in the cloud, are highlighted in this article section. Research gaps in previous research were also evaluated through various comparative analyses.

### Optimization methods based solutions

VM fusion offered an in-depth evaluation and analysis of the job based on the latest research on load balancing. A researcher mainly aims at a pre-emptive adaptive VM consolidation across cloud data centers, and their findings showed significant discoveries. A load-balancing method is essential in addressing the relationship between cloud resources and performing effective resource utilization.

A resource-efficient and dynamic consolidation of virtual machines-based technique was developed in^10^. The proposed method was based on four algorithms created at different VM fusion phases. The latest solution for VM load balancing in a cloud-based data center that considers SLAs and power consumption was put forward in^11^. A VM distribution method based on a reliable basic PSO was offered after an approach for identifying the overloaded and underloaded VMs. Enhanced distribution by learning automation, based on GA and ACO knowledge, was developed in^12^ to reduce energy use. The proposed model utilizes the GA method for finding a suitable machine for a particular workload in the cloud.

An ACO workload distribution approach is presented in^13^ to integrate the VMs in a cloud-based system. The research mainly focuses on reducing energy use and improving the distribution of workloads while completing more tasks at high performance; they established lower criteria utilizing the data canter’s overall workload utilization and employed ant colony optimization to minimize the frequency of VMs movements. Research^14^ explored optimization and ML-based work for load distribution in the cloud environment. The proposed model utilizes a bee colony optimization method. In this work, an energy utilization calculation was also performed for cloud data centers using a Planet Laboratory that included many PlanetLab virtual machines with large-scale modelling configurations.

The discrete-time systems Markov chain model was put forward in^15^ to forecast prospective resource utilization. The Host's reliability framework model has the potential to be used to classify hosts according to their state more precisely. Researchers then proposed a multi-objective virtual machine positioning method to find the optimal VMs for host mappings using the dominance-based multiple-purpose Ant Bee Colony methodology.

A multiple objectives technique centered on the PSO technique for the virtual machines' allocation problem, VM-OPSO architecture, was developed in^16^. The proposed VM-OPSO utilizes the population entropy technique to optimize the Virtual Machine Platform and accelerate integration to the most effective solution whilst increasing the number and types of the provided alternatives. In^17^, it includes information about existing cost-effective methods and supports researchers in identifying the best practical way. They investigated many state-of-the-art energy-efficient algorithms from many perspectives, including design, modelling, and measurements. The same experimental parameters were used to create and analyze alternate approaches employing the Cloud-Sim software.

A GA-based approach was presented in^18^ for adaptive virtual machine migration and host placement. This method has four unique characteristics: first, it chooses locations of hosting where VMs have to be moved that have the slightest access delay, and second, it reduces the total amount of VM migration. A multi-objective combining virtual machines technique was subsequently created using an ant colony system via double thresholds, as mentioned in^19,20^. VMs are migrated to another host when the Host is overburdened or underloaded. The proposed technique used two CPU utilization metrics to determine the Host's load condition. During combining, ACO was utilized to determine which VMs and servers should be transferred simultaneously, using varied techniques depending on the Host's traffic condition. It was built on a tactical competition that included a cloud provider, and all computing devices participated.

### Deep learning-based solution

The forecasting accuracy was enhanced using neural networks with asymmetric evolution using standard adaptation^21^. The adaptive technique improves accuracy by examining the scope of possible responses from many viewpoints and applying various potential answers. In contrast to gradient-based learning methods, this reduces the possibility of becoming spotted in optimal local circumstances. To promote precise scalability operation,^22^ presented a better prediction model under a neural network. The proposed technique classified the VMs depending on how they were employed before forecasting how they would be used in future periods. The algorithm used a multilayered perceptron classification approach to achieve the above goal. Containerized cloud-based operations can use the performance-aware automated scaling for the cloud elastic design published in^23^ to dynamically allocate the resources available in response to changing demand. A flexible scaling technique that predicts future workload demands has been developed in^24^ using Convolutional neural networks and K-means that estimate the utilization of virtual machine (VM) assets, including memory and CPU. The investigation employs a Bayesian technique to forecast VM utilization of resources throughout the typical workday.

A forecasting algorithm based on a recurring LSTM neural network model was presented to predict future HTTP workloads. The suggested method uses an ANN regarding elastic accelerating and automatic deployment-related scaling. In^25^, a multi-objective adaptive algorithm was used to estimate both memory and CPU utilization in addition to the consumption of energy for the next time slot. The researchers of^26^ offered an approach for forecasting the cloud data center amount of work. The forecasting method discussed in the article was constructed by using an LSTM network. Forecasting data stored in the cloud center demand has been suggested in^27,28^. The forecast made in the article was constructed on a network with LSTM.

The article^29^ addresses the issue of load estimation in cloud data centers. The LSTM network has been used to create the computational load forecasting model. Based on the findings from the experiment, the suggested approach has greater accuracy in forecasting than the other approaches that were also considered. In addition, many cloud service providers utilize user-defined resource criteria to offer auto-scaling features, limiting the ability to build models depending on various workload factors.

### Hybrid methods

Adapting a specific workload sequence is typically straightforward for an estimation framework that employs only one predicting model; however, it is difficult with data from the real world, while the workload pattern varies quickly over time^30^. These situations continue despite excessive capacity and under-provisioning. Two internet-based algorithms for collaborative learning^31^ were invented to predict their workloads. Models respond quickly to alterations in the cloud load request trend. An innovative cloud load forecasting method^32^ was developed for dealing with continually altering workloads. It employs numerous predictors to construct a combination of models capable of correctly anticipating real-world loads. Clouds Intelligence, an algorithm for predicting loads of work, was built with the help of several forecasts^33^.

To increase the precision of forecasts, it incorporates seven unique predicting algorithms across the domains of statistical analysis of time series, linear regression, and artificial intelligence. For forecasting a server workload pattern in a cloud-based storage center, a cloud load prediction based on a weighted fractal support vector machine algorithm is presented^34^. In this study, parametric optimization using a method called particle optimization technique was created. A different approach^35^ focuses on predicting mega-variant resource consumption in cloud centers' data. These resources comprise bandwidth for the network, processor, and storage. The method indicates resource usage using CNN and LSTM models. In the start phase, the linear connections with the mega-variant data are filtered using the matrix auto-regression approach.

Limiting the number of physical machines (PMs) that are actively processing data was the primary emphasis of previous methods. Problems with energy usage in VM provisioning and load variability are seldom addressed together. A new approach to virtual machine allocation and implementation, AFED-EF (Adaptive energy-aware VM allocation and deployment mechanism), was suggested by^36^ for use in IoT applications to address these issues. When it comes to virtual machine allocation and placement, the recommended approach performs well and can effectively manage load fluctuations. Employing a real-world workload consisting of over a thousand PlanetLab VMs, the author conducted a thorough experimental study. When compared with additional energy-aware methods, AFED-EF performed better in terms of performance, SLA violations, and overall energy usage.

To address quality of service and SLA concerns in an SDDC operating in a CAV environment, another article^37^ introduces an energy-efficient virtual machine cluster placement technique called EVCT. Using a weighted directed network as a model, the EVCT method makes use of VM similarity to solve the VM deployment issue. Using the “maximum flow and minimum cut theory” to reduce the graph into directed segments while accomplishing high energy-efficient positioning for VMs, EVCT takes traffic across VMs into account. Improved consumption of energy costs, improved scalability, and outstanding level of service for consumers are all benefits of the suggested approach. The authors also conducted a number of tests to test how well the EVCT handled a real-world load.

The issue of lowering the cloud data center’s excessive consumption of energy while limiting SLA breaches is discussed in^38^. Existing methods for managing energy resources in cloud data centers primarily aim to reduce power use, even if there are several alternatives. In order to maximize energy efficiency while decreasing power consumption, the author of this research suggests two new adaptive algorithms that take this into account. Cloud data center SLA, and violation rate analysis, are also covered. The proposed energy-aware algorithms include application kinds in addition to CPU and memory resources when deploying virtual machines, which is different from the current methods. According to the testing findings, the suggested methods may successfully reduce energy usage within cloud data centers as they maintain low SLA violations, outperforming the current energy-saving solutions.”

Table 1 presents a comparative review of existing strategies in cloud performance enhancement. It was discovered that the previous works could not simulate and anticipate workload requirements for numerous VMs. Because they were developed and educated for only one virtual machine (VM) workload, the idea that VMs are independent and contain associated applications requiring several VMs' capacity is rejected.

**Table 1:** Review of existing methods for cloud computing.

References	Methods	Predication resource	Key points	Dataset	Performance criteria
^1^	Bidirectional LSTM	VM based	VMs workload distribution on various time series data	GWAT- 12 and 13	Precision, memory utilisation
^6^	CNN with LSTM	VM based	Overcome noise in the data and workload	GWAT dataset	Accuracy, precision, utilization of resources, i.e., network, storage, memory
^10^	GRU with CNN	PM based	Overcome migration of load, energy	Telecom dataset	CPU utilisation, precision
^17^	PSO with SVM	VM based	Energy uses and workload balancing	Google cluster	Accuracy, precision, utilization of resources, i.e., network, storage, memory
^19,20^	PSO-DBN	PM based	Overcome noise in the data, workload	GWAT- 12 and 13	Accuracy, precision, memory utilization
^39^	Learning automata	PM based	Overcome migration of load, energy	CO-Mon dataset	Precision, accuracy, F-measure and memory utilization
^23^	Ensemble learning methods with PSO	VM based	Energy uses and workload balancing	Planet Lab dataset	CPU utilisation, precision
Proposed Model	DPSO-GA (Deep learning with POS-GA)	VM based	Overcome workload; reduce overloading and resource utilization	Google cluster dataset	Accuracy, precision, and utilization of resources, i.e., network, storage, and memory

## Materials and methods

This section covers the materials and methods related to the present research.

### GA method

The optimization approach known as GA is frequently employed in complicated and massive systems to determine results near the optimal level. Consequently, GA is an excellent technique for training a neural network model for learning. A standard GA is based on a population search method influenced by the process of natural selection that relies on the concept of persistence of the healthiest^40^. GA’s primary components are (a) chromosome, (b) selection process, (c) mutation process, (d) crossover, and (e) calculation and evaluation of fitness function.

We start by arbitrarily initializing a population of chromosomes, which we typically consider as potential alternatives to scheduling for any specific task. The allocation of activities to certain machines inside that chromosome allows us to obtain a fitness value (Makespan), which is acquired. After receiving the initial population, we assess each chromosome in the group according to its unique fitness value.

A smaller makespan is always desired to fine-tune the mapping. We use an allocation scheme that statistically replicates a specific chromosome and eliminates others. At the same time, we discover that improved mappings are more likely to be repeated in future generations. At the same time, the number of individuals stays constant over each age. Algorithm 1 presents the working of the GA method^41^.

**Figure:**
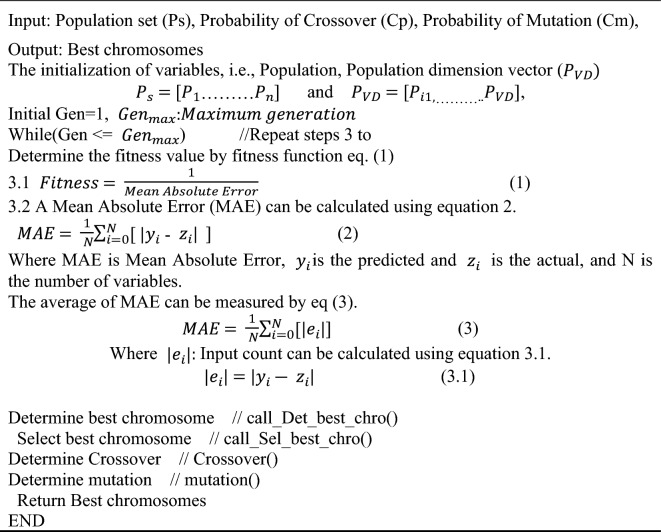
**Algorithm 1** GA algorithm

### PSO method

The swarm intelligence subcategory of optimization algorithms includes the renowned PSO algorithm. There are numerous scenarios within the literature in which PSO is used to train neural network algorithms effectively. The method comprises several particles that analyze potential solutions across the issue space, eventually arriving at the optimal ones^42^. The detailed algorithm is presented in Algorithm 2.

**Figure:**
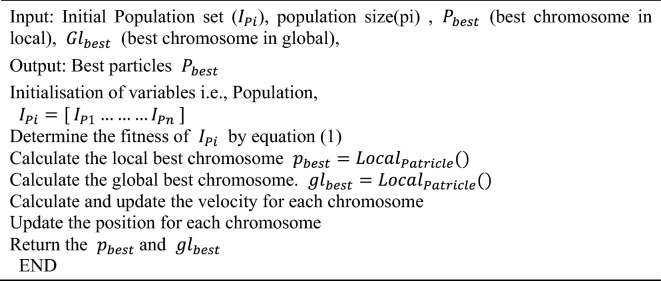
**Algorithm 2** PSO algorithm

### CNN model

**Figure 1:**
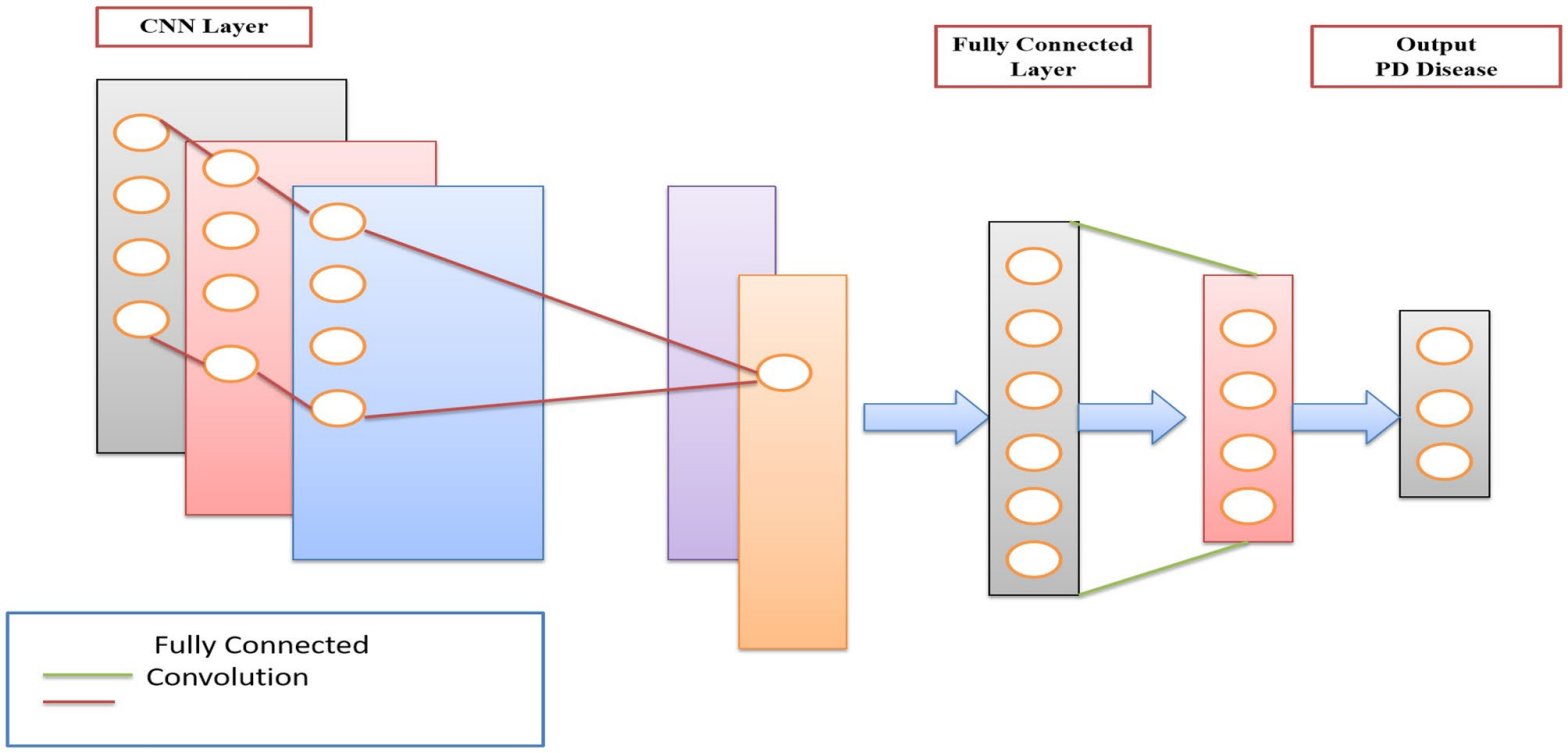
The basic architecture of the CNN model.

Deep learning techniques rely heavily on artificial neural networks (ANNs). Recurrent neural networks (RNNs), which take input as a sequence or periodic information, are a particular kind of ANN. A different type of neural network called a CNN may find crucial details in time series and visualize inputs. It is essential for data analysis, such as object and image classification. The CNN model contains three necessary layers (Convolution: 1, Pooling: 2 and Fully Connected: 3). Figure 1 presents the architecture of the Basic CNN model^43^.

Deep learning techniques rely heavily on artificial neural networks (ANNs). Recurrent neural networks (RNNs), which take input as a sequence or periodic information, are a particular kind of ANN. A different type of neural network called a CNN may find crucial details in time series and visualize inputs. It is essential for data analysis, such as object and image classification. The CNN model contains three necessary layers (Convolution: 1, Pooling: 2 and Fully Connected: 3).

### LSTM model

**Figure 2:**
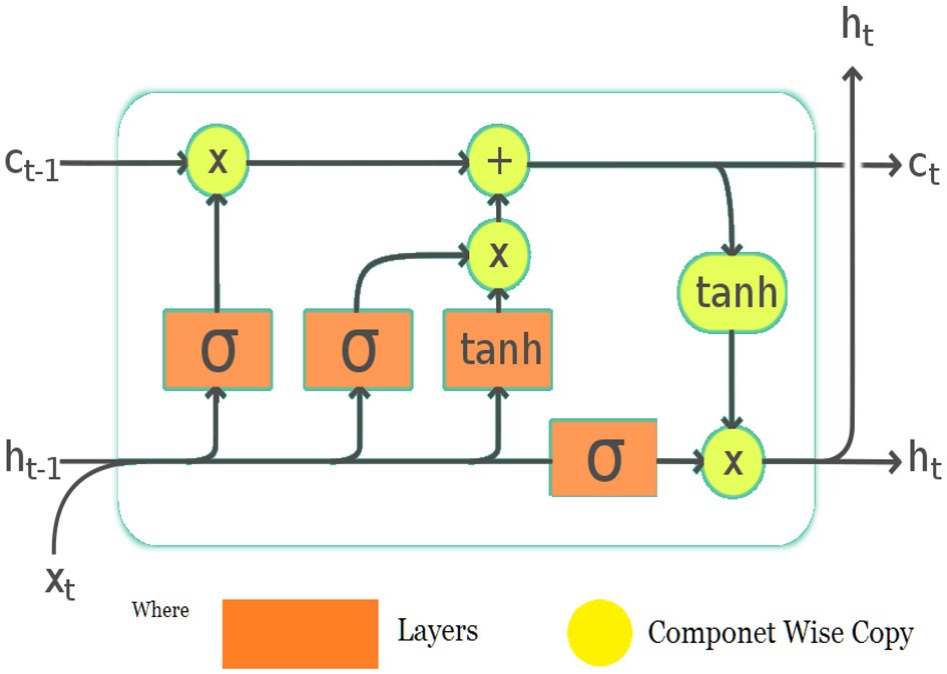
The basic architecture of the LSTM model.

It mostly applies to deep learning. Several RNNs possess the capacity to learn long-term connections, particularly in tasks involving sequence anticipation. Aside from singular observations like images, LSTM includes feedback links, making it suited to interpreting the complete data sequence. It uses automatic translation and the recognition of objects. A unique version of RNN called LSTM exhibits outstanding reliability on various issues. Figure 2 shows the basic architecture of the LSTM model^44,45^.

A memory cell, described as a “cell status” which preserves its state over time, performs a crucial part in an LSTM model. A horizontal line that travels across the top portion, as presented in Fig. 2, represents the cell’s status. It can be visualized as a network of unmodified conveyor belts across which knowledge travels. The following equations, from 4 to 6, are used to determine the computations in LSTM.

4 $${F}_{t}=\{ \sigma \left[{(W}_{F})\times \left({H}_{t-1} \right), {IX}_{t}\right]+ {B}_{F}\}$$

5 $${I}_{t}=\{ \sigma \left[{(W}_{I})\times \left({H}_{t-1} \right),{IX}_{t}\right]+ {B}_{I}\}$$

6 $${O}_{t}=\{ \sigma \left[{(W}_{O})\times \left({H}_{t-1}\right),{IX}_{t}\right]+ {B}_{I}\}$$

where ${I}_{t}$: Input Gate, ${F}_{t}$: Foregt Gate and ${O}_{t}:$ Output Gate, $\sigma :$ Sigmoid Function, ${{W}_{G}}$: Weight for a particular Gate, ${H}_{t}$: Output of the current block, ${IX}_{t}:$ Input data at present timestamp and ${B}_{G}:$ Bias value for a particular Gate.

The cell state and a candidate state with a final output can be calculated using Eqs. (7, 8, and 9).

7 $${\widehat{C}}_{{\text{t}}}=\{\mathrm{ tan}h\left[{(W}_{C})\times \left({H}_{t-1} \right),{IX}_{t}\right]+ {B}_{C}\}$$

8 $${C}_{t}=\{ \left[{({F}_{t} \times }_{ }\left({C}_{t-1} \right)\right]+ ({I}_{t}+ {\widehat{C}}_{{\text{t}}})\}$$

9 $${H}_{t}={[O}_{t}\times \mathrm{ tan}h{C}_{t}]$$

where ${C}_{t}$: Memory (Cell) state at a particular time stamp t, ${\widehat{C}}_{{\text{t}}}$: Candidate Cell state at a particular time stamp t.

### Proposed DPSOGA model

**Figure 3:**
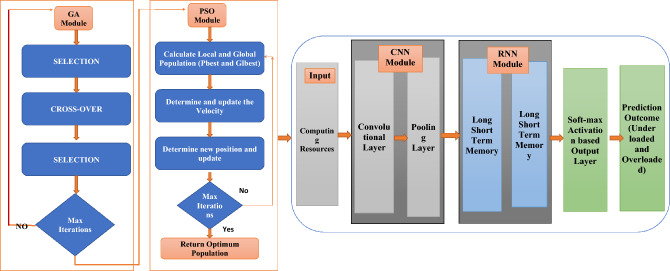
The architecture of the proposed model DPSOGA model.

To handle the workload balancing issues, this research presents Deep learning with Particle Swarm Intelligence and Genetic Algorithm based “DPSO-GA”, a Hybrid model for dynamic workload balancing in cloud computing. The proposed model simultaneously integrates the resource utilization in a multi-resource utilization, which helps overcome the load balancing and over-provisioning issues. The proposed model works in two phases^46^. The details are as follows. Figure 3 shows the architecture of the proposed model DPSOGA model.

- First phase: The first phase utilizes a hybrid PSO-GA approach to address the prediction challenge by combining the benefits of the two methods. A PSO-GA method also helps to fine-tune the Hyperparameters. In this phase, a dynamic decision-making method called the PSOGA is suggested for investigating the goal of the distribution of resources strategy. The PSO method helps to fine-tune the Hyperparameters, adjust their values automatically, and select which parameters to encode as a particle.
- Second phase: In the second phase, CNN-LSTM is utilized with PSO-GA. Before using the CNN-LSTM Approach to forecast the consumption of resources, a hybrid approach, PSO-GA, is used for training it. In the proposed framework, a one-dimensional CNN and LSTM are used to forecast cloud resource utilization at various subsequent time steps. The LSTM module simulates temporal information that predicts the upcoming VM workload, while a CNN module extracts complicated distinguishing features gathered from VM workload statistics.

In the proposed framework, a one-dimensional CNN and LSTM are used to forecast the CPU utilization on cloud-based servers at various subsequent time steps. The LSTM module simulates temporal information that predicts the upcoming VM workload, while a CNN module extracts complicated distinguishing features gathered from VM workload statistics. The CNN-LSTM module extracts the relevant components to measure the CPU usage on each cloud server at different time intervals by using connected CNN. The LSTM model keeps the temporal data, which helps reduce information loss and predict the upcoming load. The CNN layer automatically extracts the pattern information. The order of features is learned once again at the LSTM level. The proposed model continuously tunes Hyperparameters according to the results from learning CNN and LSTM^47^.

This module is responsible for finding overloaded and underloaded machines. Before introducing the novel paradigm, we evaluate the conventional VM integration architecture design. The VM integration architecture proposal involves a data center containing servers that use hybrid computing, consisting of several hosts operating different programs across multiple VMs within the information center. Each physical and virtual machine has variables, including CPU processing power, memory disc storage, and network bandwidth.

The functions Calculate_CPU_Utlization (), Call Calculate_RAM_Utlization (), Call Calculate_BW_Utlization (), and Call Calculate_Storage_Utlization () help to determine the current status of the ith machine to predict the overloaded machines. The distinctive aspects of such resource calculations are standardized individuals through a zero to one frequency. High utilization is indicated by a value nearest to 1, while low utilization is characterized by a value closest to 0. It removes the uncertainty in calculating different threshold levels in previous approaches^48^.

Equations (10–14) present the formulas^49^ for a variety of operations over CPU, memory and BW utilization using Calculate_CPU_Utlization (), Call Calculate_RAM_Utlization (); Call Calculate_BW_Utlization (), Call Calculate_Storage_Utlization ().

10 $${{\text{Calculate}}}_{{{\text{CPU}}}_{{\text{Utilisation}}}}=\frac{\sum Currently\_Running\_Task}{Total\_capacity}*100$$

11 $${{\text{Calculate}}}_{{{\text{RAM}}}_{{\text{Utilisation}}}}=\frac{\sum {{\text{RAM}}}_{\_{\text{Current}}\_{\text{Utlization}}}}{{\text{Total}}\_{\text{RAM}}}*100$$

12 $${{\text{Calculate}}}_{{{\text{BW}}}_{{\text{Utilisation}}}}=\frac{\sum {{\text{BW}}\_{\text{Current}}\_}_{{\text{Utilisation}}}}{{\text{Total}}\_{\text{RAM}}}*100$$

13 $${{\text{Calculate}}}_{{{\text{Storage}}}_{{\text{Utilization}}}}=\frac{\sum {{\text{Storage}}\_{\text{Current}}\_}_{{\text{Utilization}}}}{{\text{Total}}\_{\text{Storage}}\_{\text{Capacity}}}*100$$

14 $${{\text{Resource}}}_{{{\text{Utilization}}}_{{\text{score}}}}={\sum }_{{\text{k}}=0}^{{\text{n}}} {{\text{Calculate}}}_{{{\text{CPU}}}_{{\text{Utilization}}}}+{{\text{Calculate}}}_{{{\text{RAM}}}_{{\text{Utilization}}}}+{\mathrm{ Calculate}}_{{{\text{BW}}}_{{\text{Utilization}}}} +{{\text{Calculate}}}_{{{\text{Storage}}}_{{\text{Utilization}}}}$$

#### Overloaded and underloaded machines predication

Existing overloaded server recognition approaches are unreliable because they concentrate primarily on standard characteristics, including processor and memory usage. We introduced the various resource-conscious congested host identification approaches, which utilize a wide range of computing resources to determine whether a server/VM is overloaded. It also determines Memory usage, RAM and storage utilization, network traffic and bandwidth consumption, and storage capacity.

It is the primary instance when arrays of computing resources are used as parameters in an integrated form to forecast the overloaded machines in the cloud environment. Using different resources improves the efficiency and reliability of the VM integration architecture. All accessible cloud machines and servers are classified into two classes: loaded and overloaded. Any devices that are currently overloaded remain passive or overwhelmed. The approach we suggest is a fusion of the CNN and LSTM methods, where we utilized an appropriate weight control method that normalized the hosts' capabilities towards a usual spectrum of zero to one. It enhances the system's functioning and dependability and offers an adaptive overloading recognition approach.

We proposed an additional resource awareness underloaded server/Machine (VM and PM) recognition approach to boost VM placement effectiveness and decrease migration frequency without jeopardizing SLA compliance. The proposed underloaded recognition mechanism separates each of the three categories of machines. A resource utilization score helps to decide whether the host/machine is overloaded or under load; we are considering four classes: Idle load (IL), Overloaded (OL), Underloaded (UL) and Free Host (FH). It exhibits a relatively simple and efficient strategy that eliminates the tedious and complicated procedure of finding numerous threshold levels. The proposed method achieves flexibility and dynamic processes by normalizing every measurement. Algorithm 3 presents the working of CNN-LSTM fusion with PSO-GA for overloaded and underloaded machine prediction.

**Figure:**
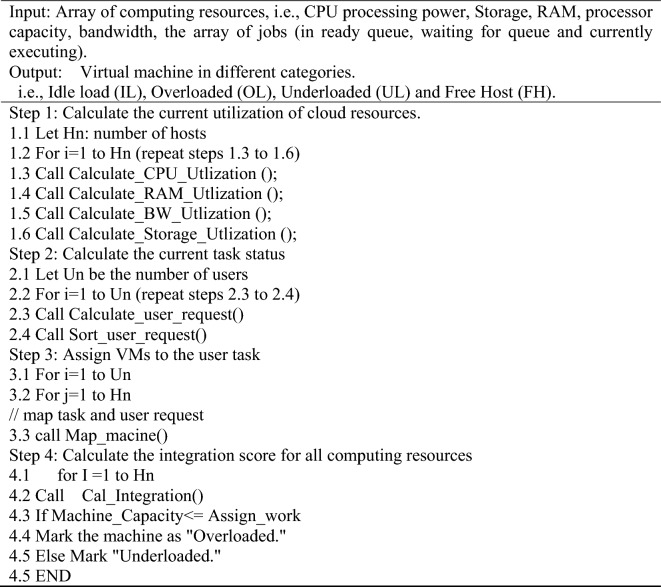
**Algorithm 3** To predict the overloaded machine in the cloud environment

#### VM selection

When overburdened servers are noticed, the approach attempts to determine the servers and VMs that must be moved from a particular host to a different one via predefined VM deployment strategies. An additional virtual machine has been chosen if the server is still overburdened. The proposed model utilizes a PSOGA-based method for VM selection, which operates a Lowest_Migration_Time () to select the appropriate machine. Algorithm 4 defines the VM selection process.

**Figure:**
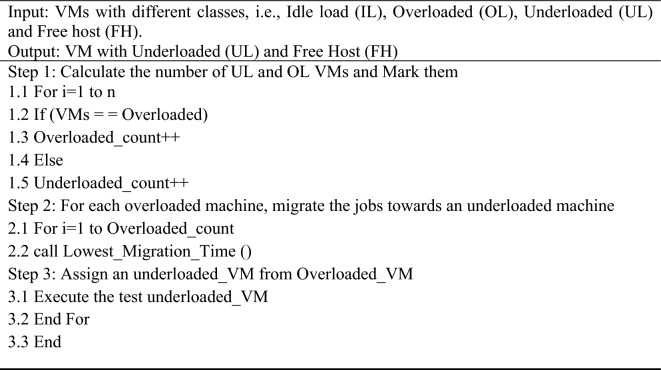
**Algorithm 4** To select the correct VM machine

### Performance measuring parameters

The following parameters are used for performance comparison between the proposed and existing methods. Each parameter is calculated separately for both methods (proposed and existing). Performance metrics for the cloud scheduling algorithms are based on the following factors-.

- Average waiting time: It is defined as how long each process has to wait before it gets its time slice.
- Average response time: It is the time taken from when a process is submitted until the first response is produced^15^. Average response times for each algorithm have decreased by increasing the number of CPUs.
- Makespan: It can be defined as the overall task completion time. We denote the completion time of task T_i_ on VM_j_ as CT_ij_.
- Energy Consumption: The sum of energy consumed by PMs. A linear cubic energy utilization approach determines PMs’ energy utilization.
  15 $${{\text{E}}}_{{\text{c}}}={{{\{\mathrm{ E}}_{{\text{c}}}^{{\text{Idle}}}}^{ }}_{ }+ \left[{\mathrm{ E}}_{{\text{c}}}^{{\text{max}}}-{\mathrm{ E}}_{{\text{c}}}^{{\text{idle}}} \right]*{\mathrm{ U}}_{{\text{c}}}^{3}$$
  where ${{\text{E}}}_{{\text{c}}}$: Energy consumption, ${{\text{E}}}_{{\text{c}}}^{{\text{Idle}}}$: Idle state energy, Uc: CPU utilization.
- Precision: It can be calculated by Eq. (16).
  16 $${\text{Precision}} = {\text{True}}\;{\text{positive}}/\left( {{\text{True}}\;{\text{positive}} + {\text{False}}\;{\text{positives}}} \right)$$
- Recall: It can be calculated by Eq. (7).
  17 $${\text{Recall}} = \frac{{{\text{True}}\;{\text{positives}}}}{{{\text{True}}\;{\text{positive}} + {\text{False}}\;{\text{negative}}}}$$

F-Measure: It can be calculated by Eq. (18).

18 $${\text{FMeasure}}= 2*\frac{({\text{Precision}}*\mathrm{ Recall})}{\left(\mathrm{Precision }+\mathrm{ Recall}\right) }$$

Accuracy: It can be calculated by Eq. (19).

19 $${\text{Accuracy}} = { }\frac{{\left( {{\text{True}}\;{\text{Positive}} + {\text{True}}\;{\text{Negative}}} \right)}}{{\left( {{\text{True}}\;{\text{Positive}} + {\text{True}}\;{\text{Negative}} + {\text{False}}\;{\text{Positive}} + {\text{False}}\;{\text{Negative}}} \right)}}{ }$$

## Experimental results and discussion

This section covers experimental details and results and discussion. An experimental analysis was performed in two different phases. The details are as follows.

### Phase 1 experimental analysis

The first phase uses the PSO-GA method to perform efficient load balancing. An experimental analysis was performed on a cloud-sim simulator. The existing PSO, GA and PSO-GA (proposed Hybrid) were implemented. With 10–100 VMs and 50–1000 jobs on the cloud-sim simulator, the experiments were carried out through 15 data centers. The task has between 1000 MI (Million Instructions) and 20,000 MI. Table 2: PSO-GA parameters and Table 3 presents the cloud simulator's feature configurations/parameters overview.

**Table 2:** PSO-GA parameters.

Parameter name	Value
Population used	0–100
Cross over type	Single point standard
Rate for mutation	0.06
Iterations	0–100
Executions count	0–500
acceleration coefficients one	1.0
acceleration coefficients two	1.2
fitness function	0.1–0.4

**Table 3:** Cloud sim parameters.

S no	Entity	Parameter name	Value
1	Cloud lets (Tasks)	No of tasks	100–1500
		Length of task	500–2500 MI
2	VMs (virtual machines)	No of VMs	10–100
		MIPS	500–2000
		RAM (VM memory)	250–3000
		Bandwidth	250–1500
		Cloud let scheduling method	Shared time and shared space method
		No of PEs requirements	10–50
3	Data center	No data center	25
		VMS scheduler	Shared time and shared space method
		No of hosts	20

**Figure 4:**
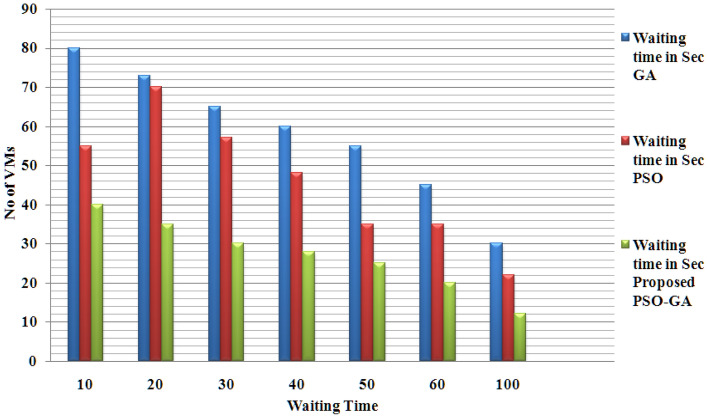
Waiting time for proposed and existing methods.

Figure 4 presents the waiting time for proposed and existing methods. Waiting time is calculated for various virtual machines from 10 to 100 with different capacities for all three methods. That proposed method shows better waiting time results than existing PSO and GA methods.

**Figure 5:**
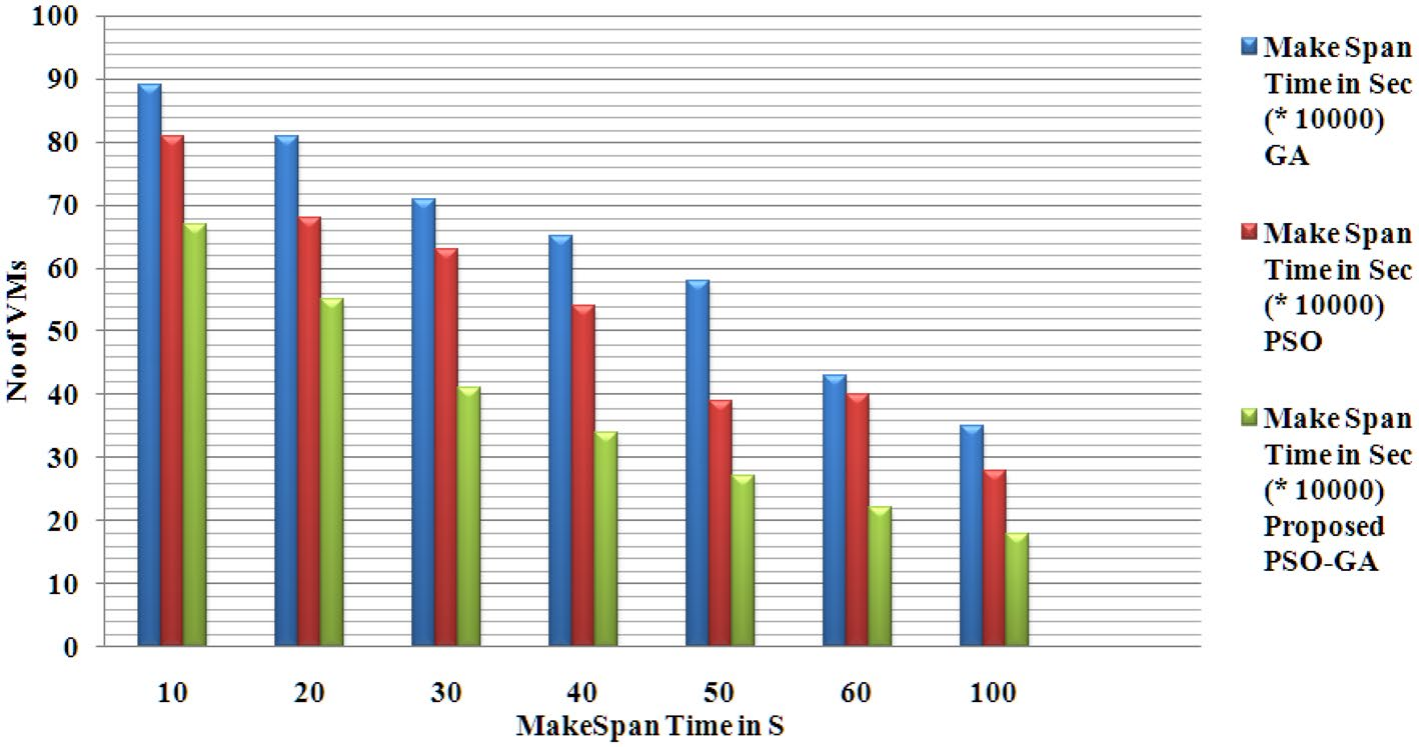
Makespan time for proposed and existing methods.

Figure 5 presents the makespan time for the proposed and existing methods. Makespan time is calculated for various virtual machines from 10 to 100 with different capacities for all three methods. That proposed method shows better makespan time results than existing PSO and GA methods.

**Figure 6:**
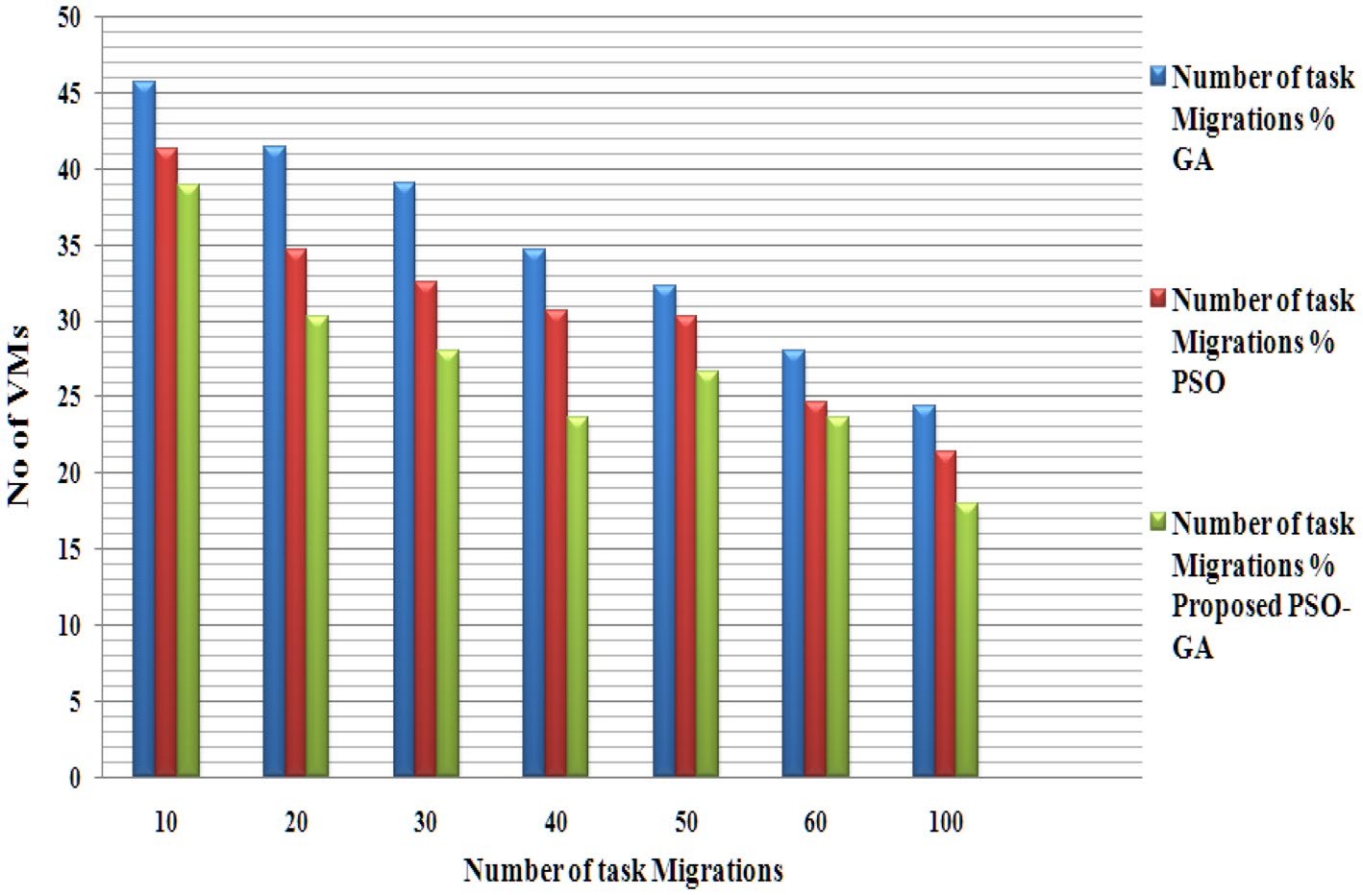
No task migrations.

Figure 6 presents the number of task migrations for proposed and existing methods. Fewer task migrations show a better performance. The task migrations in the proposed model are minimal due to extensive dynamic workload prediction and GA fusion with PSO; it helps identify the most appropriate VM for each job.

Table 4 presents the running time of algorithms using existing GA, PSO, and the proposed hybrid PSOGA method. The analysis was performed using the number of tasks from 50 to 1500. The proposed model achieved better running time over existing GA and PSO methods.

**Table 4:** Running time (in s) analysis results.

Technique	Number of tasks				
	50	100	500	1000	1500
GA	0.8142	1.879	17.895	20.741	22.452
PSO	0.764	1.457	16.451	18.778	19.997
PSOGA	0.712	1.231	15.442	17.481	18.651

**Figure 7:**
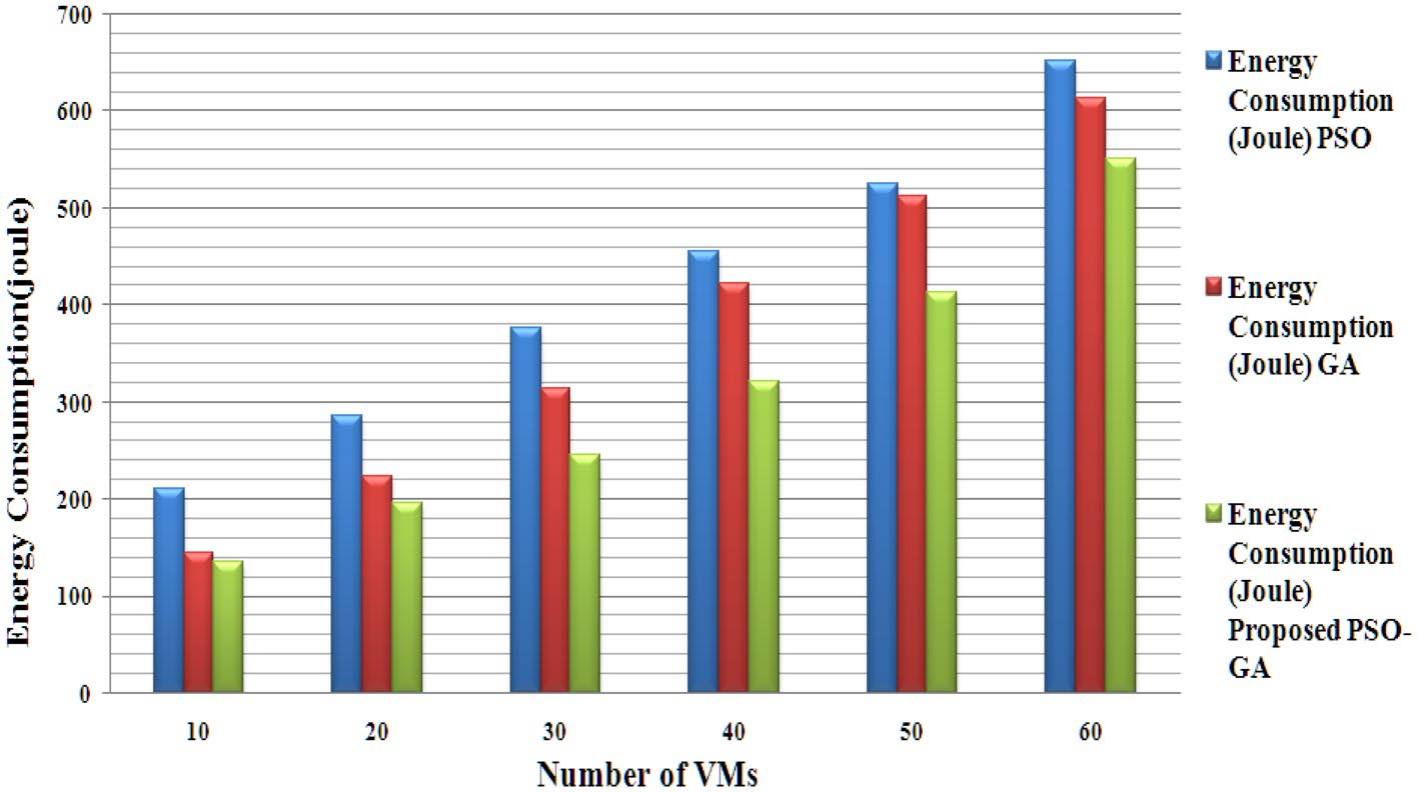
Energy consumption based on VMs.

Table 5 presents Energy consumption results (KWh) based on the number of tasks for 100 VMs, and Fig. 7 illustrates the graphical comparison of energy consumption results PSO, GA, and Proposed method based on VMs. The proposed model consumes less energy than existing methods.

**Table 5:** Energy consumption REsults (KWh) (based on the number of tasks for 100 VMs).

Technique	Number of tasks				
	50	100	500	1000	1500
GA	223.84	279.36	398.96	647.32	889.95
PSO	209.31	245.63	378.24	631.78	881.23
PSOGA	201.77	225.91	351.40	611.78	809.91

### Phase two

The second phase utilizes the online Kaggle datasets “Google cluster workload traces 2019”^47^. The trace mainly contains the complete details for each task, i.e., obedience, schedule preference, and resource information consumption for the assignments executed within these clusters. We compare the DPSOGA proposed model (CNN-LSTM with PSO-GA) and the existing CNN LSTM model.

Each operation within the data set is comprised of numerous continuous assignments which are executed on different systems. The dataset includes CPU and memory utilization, disc usage, etc. Prior studies^1,2^ demonstrated that less than 3% of operations need an extended period. A lengthy job containing ID “Job-16-17658948” and 62,071 procedures previously utilized in examinations had been selected to assess proposed and existing models.

This analysis includes multi-variate analysis that considers storage, processing power and memory and uni-variate analysis that only considers limited parameters. We examined the outcomes of the proposed hybrid model (CNN-LSTM with PSO-GA) to the results of the (CNN-LSTM without PSO-GA). An “exponential linear unit method” (ELUM) is used as an activation parameter in each analyzed model’s input and output layers. The outcomes of the proposed hybrid model’s assessment compared to alternative approaches concerning “mean absolute error” (MAE) are displayed in Table 6.

**Table 6:** Simulation results for MAE (existing vs. proposed).

Model	Input category	Parameters used		
		Storage	Processing power	Memory
Proposed hybrid model	Multi-variate analysis	0.18	0.29	0.024
	Uni-variate analysis	0.12	0.21	0.017
Existing model	Multi-variate analysis	0.25	0.37	0.036
	Uni-variate analysis	0.17	0.25	0.029

**Figure 8:**
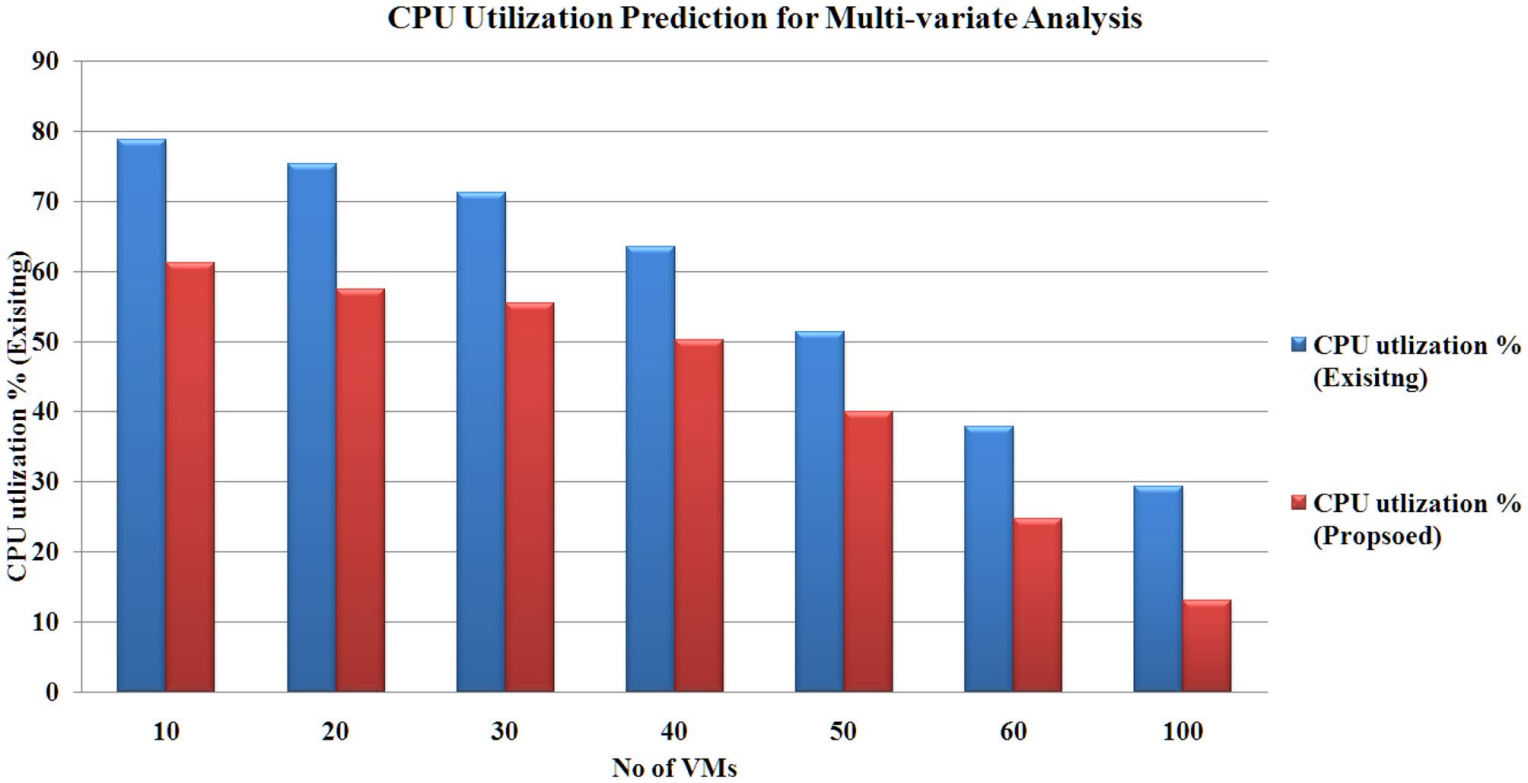
CPU utilization % results for multi-variate analysis.

**Figure 9:**
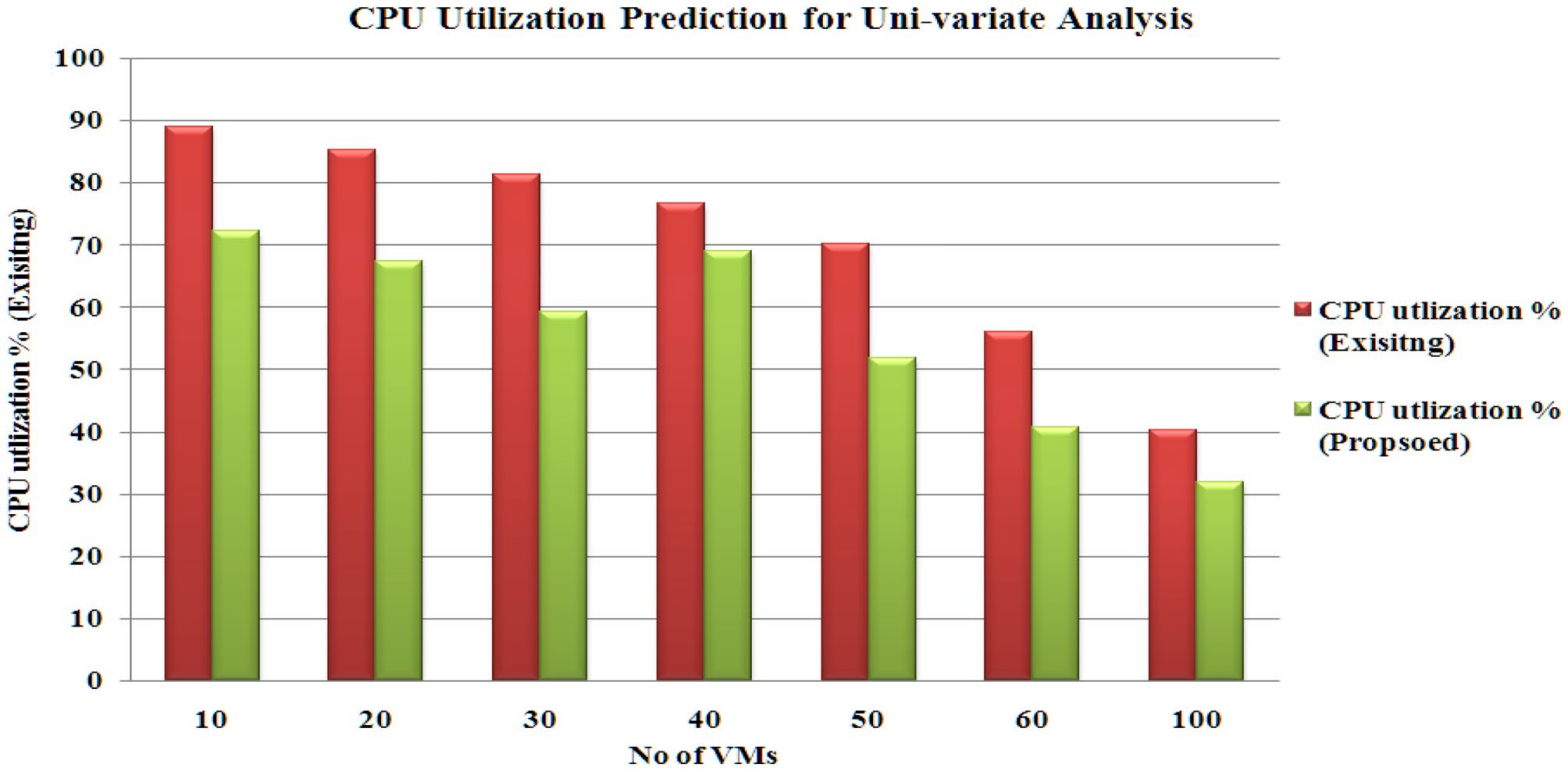
CPU utilization % results for uni-variate analysis.

**Figure 10:**
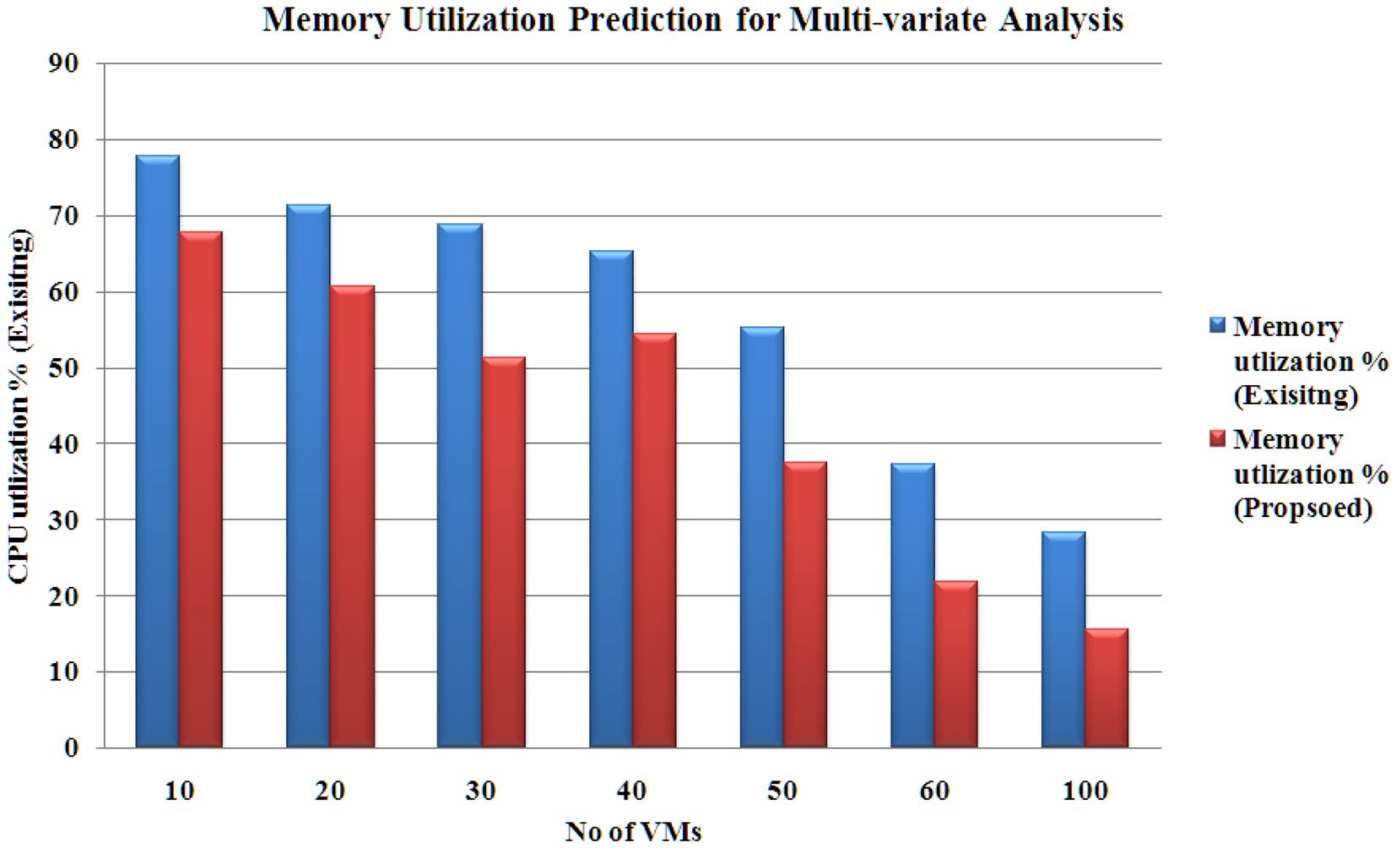
Memory utilization % results for multi-variate analysis.

**Figure 11:**
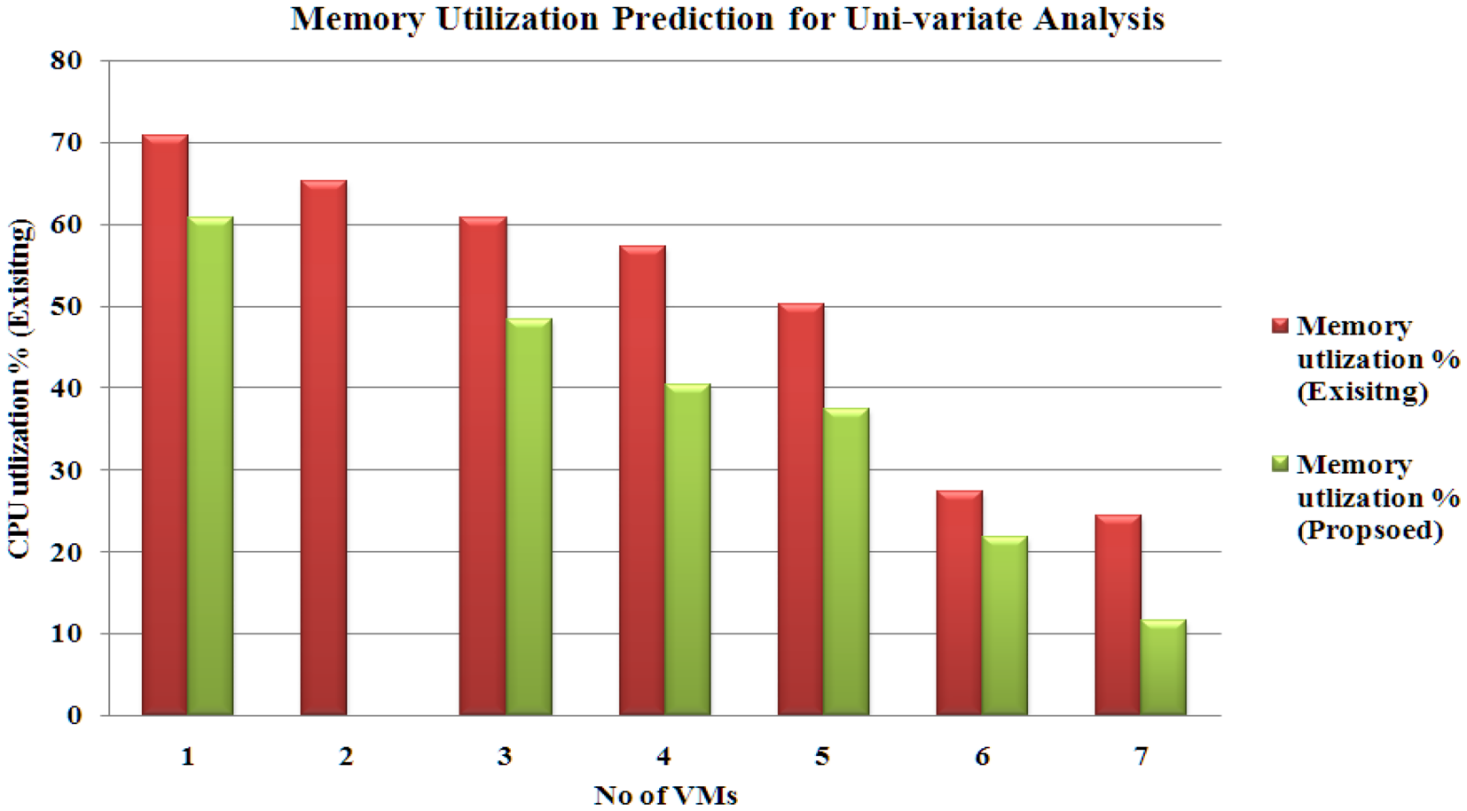
Memory utilization % results for uni-variate analysis.

The outcomes are calculated using a ‘sliding window’ with dimension 5. The results prove that the proposed hybrid model (CNN-LSTM with PSO-GA) produces reduced products under uni-variate and multi-variate input scenarios. Figures 8, 9, 10 and 11 present the various outcomes of the analysis of the existing proposed model on multi-variate and uni-variate feature sets.

### Results and discussion

To overcome cloud load balancing and high provision of computing resources issues, this research presented a “DPSO-GA”, a Hybrid model for dynamic workload balancing in cloud computing. The complete analysis is divided into two phases to accomplish the research objective. The first phase is based on PSOGA, and the second phase utilizes CNN-LSTM with PSO-GA. The scheduling of tasks in cloud-based systems was addressed in the first phase by the PSOGA algorithm, which was conceived and developed using the Cloud-sim simulation. The proposed method's efficiency was compared with well-known algorithms like GA and PSO.

Figures 4, 5 and 6 present the results of the phase 1 simulation. The PSOGA method's architecture enables task execution across VMs via an equitable workload distribution among fast and slow VMs, avoiding overloading any VM over the others. As opposed to consuming the fast VMs and delaying the job performance overall, this strategy decreases the Makespan by using the slow VMs relatively. Table 2 presents the PSO-GA parameter setting, and Table 3 shows Cloud Sim parameters. The proposed method (PSO-GA) achieved a better waiting time of 10.2 s for 10 VMs, which is better than the existing PSO and GA. A Makespan time presents the utilization of VMs, and less Makespan time shows a better performance. The proposed PSO-GA achieved the lowest makespan time for 100 VMs and less migration time, delivering better performance than PSO and GA. Similar to Table 4, the number of tasks running time was calculated. The proposed method achieved 0.712 running times (in a s) for 50 studies and 18.651 (s) for 1500 tasks, which is better than the existing method. Infusion of PSO-GA enhances the overall performance of the model.

In experimental two, the proposed model performs better than the existing hybrid model (CNN-LSTM without PSO-GA). PSO-GA model was used to train the CNN-LSTM model. Table 6 presents an MAE analysis for experiment 2 for the proposed and existing method. In the proposed method, a PSO-GA method was used to train the CNN-LSTM model, which helps to enhance the model's performance. For Multi-variate analysis proposed model achieved MAE results Storage: 0.18, Processing power:0.29 and Memory:0.024, and similar to Uni-variate research proposed model acquired MAE results Storage: 0.12, Processing power:0.21 and Memory: 0.017, which is far better than the existing model which achieved For Multi-variate analysis proposed model achieved MAE results Storage: 0.25, Processing power: 0.37 and Memory:0.036, and similar for Uni-variate analysis proposed model earned MAE results Storage: 0.17, Processing power:0.25 and Memory:0.029. Figures 8, 9, 10 and 11 shows the CPU, memory, and storage utilization % results for Multi-variate and Unvaried analysis. The proposed method performs outstandingly in displaying less resource utilization and task migration.

### Time complexity analysis of proposed method

The two main parts of the proposed technique in this paper are the particle and velocity initialization and the updates to the locations and velocities of the particles, as well as the assessment of fitness solutions for PSO and GA. Finding the total time required to execute the method is the first step in calculating its time complexity.

Assume s is the size of the population, v is the size of the virtual machines, and c is the number of tasks associated with the submitted requests. During mass initialization, all of the masses in the population are given random placements and velocities. The fitness of the present mass location is determined at startup. Performing mass initialization has a temporal complexity of [O(v × c)]. Thus, initializing the whole population has a temporal complexity of O(s × v × c). The iteration loop begins by updating the global variables. The number of steps required to complete that activity is [O(s_3_ + (s × c))]. Iteratively gathering the highest and lowest fitnesses from the whole swarm is necessary within update Global Variables, which is why the process takes so long. Such operations have an [O(s)] time complexity. We also determine the total forces acting on each mass and their acceleration inside this procedure. It takes [O(s3 + (s × c))] seconds to complete those activities.

Iteratively updating global variables also has this temporal complexity. Secondly, for every mass in the population, a loop is iterated. We are updating the location and velocity of each group. Both of these updates have a temporal complexity of [O(v × c)]. Revising the mass's fitness is the next stage. It takes [O(v)] steps to complete the operation. The particle loop has an overall temporal complexity of [O(s × v × c)]. Therefore, the temporal complexity for the iterations loop is [O((s × MAX_ITERATION) × (s2 + (v × c)))]. Last but not least, switch back the mapping from cloudlet to virtual machine. This action has a temporal complexity of O(v × c). The total time complexity, after adding up all the steps' time complexity, is [O(s × MAX_ITERATION × (s2 + (v × c))).

## Conclusion and future work

In cloud computing, load balancing plays an essential role in the performance improvement of the entire system. Cloud computing technology offers various opportunities and services for using IT infrastructure as a utility with many possibilities, like scaling down and scaling up, depending upon the organization’s needs. However, like most rising technologies, cloud computing also has issues, i.e., high provision of computing resources, load balancing and energy consumption, that must be resolved. To overcome these issues, this research presents a “DPSO-GA”, a Hybrid model for dynamic workload balancing in cloud computing. The proposed model works in two phases. The first phase utilizes a hybrid PSO-GA approach to address the prediction challenge by combining the benefits of the two methods. A PSO-GA method also helps to fine-tune the Hyperparameters. In the second phase, CNN-LSTM is utilized. Before using the CNN-LSTM Approach to forecast the consumption of resources, a hybrid approach, PSO-GA, is used for training it.

The simulation results of the first phase include waiting time, task migration time, response time and task running time. The proposed PSOGA fusion performs better than the GA and PSO methods. In the second phase, comprehensive simulations are carried out utilizing the Google cluster traces benchmarks dataset to verify the efficiency of the proposed DPSO-GA technique in enhancing the distribution of resources and load balancing for the cloud. This research can include multiple data centers in a diverse setting. Additionally, the job can be improved by applying dynamic workflow, which gives clients more flexibility to modify the attributes of workflow activities as they are being performed. Numerous parameters make up the scheduling issue, yet some conflict. While enhancing the ideal variables, the developed methods must consider the repercussions of other factors. Furthermore, factors such as privacy and security must be addressed. Applying the modelled algorithm in real-world settings can also present difficulties, including costs associated with administration, energy used for purposes other than computation, hardware problems, and data transfers.

## Data Availability

The datasets used in the current research are available from the corresponding author upon individual request.
